# Genome Sequences of Five Additional *Brevibacillus laterosporus* Bacteriophages

**DOI:** 10.1128/genomeA.01146-15

**Published:** 2015-10-22

**Authors:** Bryan D. Merrill, Jordan A. Berg, Kiel A. Graves, Andy T. Ward, Jared A. Hilton, Braden N. Wake, Julianne H. Grose, Donald P. Breakwell, Sandra H. Burnett

**Affiliations:** Department of Microbiology and Molecular Biology, Brigham Young University, Provo, Utah, USA

## Abstract

*Brevibacillus laterosporus* has been isolated from many different environments, including beehives, and produces compounds that are toxic to many organisms. Five *B. laterosporus* phages have been isolated previously. Here, we announce five additional phages that infect this bacterium, including the first *B. laterosporus* siphoviruses to be discovered.

## GENOME ANNOUNCEMENT

*Brevibacillus laterosporus* is a spore-forming *Firmicutes* bacterium that is found in many locations, including beehives (1). This bacterium is a secondary invader following European Foulbrood of honeybees (2). It also produces compounds that are toxic to many organisms including bacteria, fungi, nematodes, mollusks, and mosquitoes, making it potentially useful for bioremediation or biocontrol (1, 3–6). Five phages infecting this genus have been described previously (7). We announce five additional complete genome sequences of bacteriophages that infect *B. laterosporus*.

These phages were isolated by enrichment of bee debris samples gathered from beehives in Utah using field isolates previously described as being *Paenibacillus larvae*. Phylogenetic analysis of the 16S rRNA gene sequences indicates these field isolates, as well as *P. larvae* subsp. *pulvifaciens* DSM 8442 and DSM 8443 are indeed *B. laterosporus* bacteria. This finding was further supported by PCR and sequencing of additional loci. Phages were isolated and then plaque purified using either BL2, BL6, or BL14 as described previously (7, 8). Transmission electron micrographs were obtained for each phage to determine structural morphotypes. Phage DNA was extracted (Norgen Biotek, Thorold, ON) following manufacturer specifications. Osiris, Jenst, and SecTim467 were sequenced using 454, while Powder and Sundance were sequenced using Illumina. Sequences were assembled using Newbler 2.9 (Roche Diagnostics, Branford, CT) and Consed (9). Analysis of raw sequencing data, read pileups (PAUSE, https://cpt.tamu.edu/computer-resources/pause/) and large terminase proteins indicates that Powder and Osiris are circularly permuted, while Jenst, SecTim467, and Sundance have short direct terminal repeats. Phages were annotated using DNA Master as described previously (7). Phages Osiris, and Powder exhibited a myovirus morphology, their genomes contained 103 open reading frames (ORFs) and lacked coding for any tRNA sequences. Phages Jenst, SecTim467, and Sundance exhibited a siphovirus morphology and their genomes contained 178, 183, and 194 ORFs, respectively, with Jenst and SecTim467 containing 6 tRNAs and Sundance lacking tRNAs.

These five phages, as well as the five phages described previously (Jimmer1, Jimmer2, Abouo, Davies, and Emery) (8) were able to infect *B. laterosporus* BGSC 40A1 (10), suggesting that all ten are phages of *B. laterosporus* and not of *P. larvae* as previously reported. These ten phages represent the first phages isolated that infect the genus *Brevibacillus*. 

### Nucleotide sequence accession numbers.

GenBank accession numbers for the five *Brevibacillus laterosporus* bacteriophages are listed in Table 1.

**TABLE 1:** *Brevibacillus laterosporus* bacteriophage genomes

Phage name	Accession no.	Fold coverage	Genome length	G+C content (%)
Osiris	KT151956	351.6	52,955 bp	38.10
Powder	KT151958	73.5	52,992 bp	38.14
Jenst	KT151955	87.3	126,341 bp	42.89
SecTim467	KT151957	122.9	130,482 bp	42.71
Sundance	KT151959	20.4	134,270 bp	35.50
